# New Insight in Histamine Functions

**DOI:** 10.3390/biom12050609

**Published:** 2022-04-19

**Authors:** Silvia Sgambellone, Silvia Marri, Emanuela Masini, Laura Lucarini

**Affiliations:** Department of Neuroscience, Psychology, Drug Research and Child Health (NEUROFARBA), Section of Pharmacology, University of Florence, Viale Gaetano Pieraccini, 6, 50139 Florence, Italy; silvia.sgambellone@unifi.it (S.S.); silvia.marri@unifi.it (S.M.); emanuela.masini@unifi.it (E.M.)

The first properties of histamine (HA) that were elucidated were vasodilation and contraction of smooth muscles in the gut after stimulating gastric acid secretion and constriction of the bronchial area during anaphylaxis [1,2]. Earliest studies suggested a function in the brain, and it has been identified as neurotransmitter [3]. This Special Issue includes eight original articles and five reviews from more than 69 scientists around the world, working in the intriguing field of HA and focuses on recent knowledge regarding the involvement of HA in important physiological function and diseases.

This Special Issue begins with an article by Eissa and colleagues [4], which focuses on an important and novel role of HA in autism spectrum disorder (ASD) in an idiopathic ASD mice model, where the compound E100, a molecule with histamine H_3_ receptor (H_3_R) antagonistic affinity and acetylcholinesterase (AChE) inhibition, alleviates autistic-like behaviors and modulates disturbed anxiety levels. The overall results observed show that E100 at a dose of 5 mg/kg reduces assessed behavioral deficit and demonstrates that simultaneous targeting of histaminergic and cholinergic neurotransmission is crucial for palliation of ASD-like features; however, further in vivo research on its effects on brain levels of acetylcholine (Ach) and HA are needed.

Another interesting study on histaminergic neurotransmission was carried out by Rani and coworkers [5] on the topic of social recognition memory. The ability to recognize familiar conspecifics is essential for many forms of social interaction, including reproduction, establishment of dominance hierarchies, and pair-bond formation in monogamous species. The authors demonstrate that the disruption or the potentiation of histaminergic transmission differentially affects short- (STM) and long-term (LTM) social recognition memory. In HA-deprived animals, either chronically (HDC^−/−^) or acutely (mice treated with α-fluoro-methylhistidine (αFMH), a histidine decarboxylase irreversible inhibitor), LTM is affected, but not STM. The blockage of H3R with ciproxifan, which increases HA release, has procognitive effect, which is absent in both HDC^−/−^ and α-FMH-treated mice. The restriction of HA release with an H3R agonist impairs both STM and LTM, an effect prevented by pre-treatment with an AChE inhibitor. These observations strongly suggest that brain HA is essential for the consolidation of LTM, but not of STM, in social recognition tests.

A peculiar and interesting article is that of Rodriguez and coworkers [6] on the ingestion of HA from Anopheles mosquitoes. *Anopheles stephensi* is a vector of malaria parasites, and is an important sanitation problem in many countries; in fact, an estimated 229 million people worldwide were infected by this parasite in 2019. Individuals with severe malaria can present increased HA blood concentration, potentially delivered to mosquitoes during blood-feeding. The results indicate that HA ingestion by *Anopheles stephensi* at a level consistent with severe malaria can enhance mosquito behaviors and parasite infection success, amplifying parasite transmission to and from human hosts.

Ma and coworkers [7] identified a novel histamine H4 receptor (H_4_R) interactor. This receptor, a G protein-coupled receptor, predominantly expressed on immune cells, activates G-proteins and recruits β-arrestin upon phosphorylation by GPCR kinases to induce cellular signaling in response to agonist stimulation. In the last few decades, novel interacting proteins have been identified. In this study, the authors used a split-ubiquitin membrane yeast two-hybrid assay to identify H_4_R interactors in a Jurkat T cell line cDNA library. Using the MYTH screen, seventy interactors were identified, which interacted with other GPCR subtypes. The result confirmed using other techniques, is an important starting point to further investigate the regulation of GPCR signaling.

Wilzopolski and coworkers [8] investigated the involvement of TRPV1 and TRPA1 channels in the transmission of histaminergic itch. Two histamine receptor (HR) subtypes, namely H_1_ receptor (H_1_R) and H_4_R, are involved in the transmission of histaminergic-induced itch. The potential efficacies of two inhibitors of TRPV1 and TRPA1 channels to modulate H_1_R- and H_4_R-induced transmission were tested in an in vivo scratching assay in mice, and in vitro in murine sensory dorsal root ganglia (DRG) neurons. The results clearly indicated that both channels are involved in the transmission of HA-induced pruritus, suggesting that inhibitors of this channels could be used in the future for controlling itch.

The paper by Micheli and coworkers [9] investigated the interactions between H_4_R and adenosine A_3_ receptor (A_3_AR) in modulating neuropathic pain. A_3_AR agonists have emerged as potential relievers of neuropathic pain via T cell-mediated production of IL-10 [10]. In a mouse model of chronic constriction injury (CCI), i.p. co-administration of a A_3_AR agonist and an H_4_R agonist are effective in counteracting mechano-allodynia increasing IL-10 plasma levels. In H_4_R^−/−^ mice the analgesic effect of A_3_AR agonist is reduced and is restored after i.v. administration of CD4+ T cells obtained from naïve wild-type mice. The results indicate the role of histaminergic system in the mechanism of A_3_AR-mediated neuropathic pain relief through IL-10 upregulation.

Verta and coworkers [11] thoroughly investigated the role of histamine H4R in renal functioning in diabetic mice using healthy and diabetic H_4_R^−/−^ mice compared to their C57BL/6J wild-type counterpart. Mice, knocked out for H_4_R, showed a lower urine outflow and albumin-to-creatinine ratio compared to wild-type mice. A higher expression of megalin and a lower expression of sodium–hydrogen exchanger (NHE)3 and aquaporin was present in the H_4_R^−/−^ mice, which developed more severe hyperglycemia and a higher 24 h urine volume. These events are correlated with reduced NHE3 over-expression and megalin loss. These results highlight the role of H4R in the control of renal reabsorption processes.

The work presented by Rosa and coworkers [12] focused on the role of histamine H_3_R in the control of autonomic neuropathy associated with diabetes. Utilizing α-CNS-sparing H_3_R receptor antagonist (PF0086087) in streptozocin-induced diabetic rats, the authors investigated its effect on intestinal tract neuropathy. The drug prevents mucin production and restores the inhibitory component of enteric mobility, modified by diabetes, clearly indicating that compound PF0086087 is an essential tool for preventing nitrergic dysfunction in the myenteric plexus of the distal colon and diabetes-induced gastrointestinal complications.

A review by Nguyen and coworkers [13] investigated the pathophysiological role of HA receptor in cancer progression. High levels of HA and its receptors (H_1_R-H_4_R) are present in many different types of tumor cells and in the tumor microenvironment, suggesting a role in tumor progression. This review summarizes recent evidence and discusses a novel therapeutic approach for HA ligands and their potential prognostic values in cancer treatment. The effects of various H_1_ and H_2_ receptor (H_2_R) antagonists and H_4_R agonists on tumor progression, in many different cancer types, have been described and HA receptors (HR) subtypes may serve as prognostic biomarkers in several cancers. Taken together, HR ligands could be novel therapeutic drug for cancer treatment, alone or in combination with conventional therapy.

The review by Moya-Garcia and coworkers [14] on HA, metabolic remodeling, and angiogenesis brings together and analyzes current information on the relationship between the “histamine system” and other important metabolic processes important for homeostasis. In fact, HA metabolism confers a very complex network that connects to many metabolic processes that are important for homeostasis, including nitrogen and energy metabolism. The molecular characterization of the role of HA in the modulation of angiogenesis-mediated processes, such as in cancer, suggests an important and promising investigation field for future biomedical advances.

Novel insights into HA and its receptor ligands in terms of glaucoma and retina neuroprotection are reported in the review by Sgambellone and coworkers [15]. Glaucoma, a multifactorial neuropathy characterized by increased intraocular pressure (IOP) and/or short-term IOP fluctuation, combines a group of optic neuropathies that evolve into a progressive degeneration of retinal ganglionic cells (RGCs). This review summarizes findings from animal models on the role of histamine and its receptors in the eye, focusing on recent evidence on the effects of H3R antagonists for future treatment of glaucomatous patients [16].

The review by Comas-Basté and coworkers [17] focuses the current state of art on HA intolerance. This intolerance, referred to as enteral histaminosis or sensitivity to dietary HA, is a disorder associated with an impaired ability to metabolize ingested HA that was described at the beginning of the 21st century and, unfortunately, has grown considerably in recent years. This review provides an updated survey on HA intolerance, mainly focusing on its etiology, and existing diagnostic and therapeutic strategies.

In the final review by Cheng and coworkers [18], the authors briefly describe the distribution of HA neurons and receptors and their intracellular pathways. Moreover, the authors summarized recent experimental results and clinical findings of the role of the histaminergic system in neuropsychiatric disorders, such as narcolepsy, Alzheimer’s disease, Tourette’s syndrome, and Parkinson’s disease, providing some perspectives on the curative role of histaminergic ligands in neuropsychiatric disorders.

In conclusion, the papers in this Special Issue focus on novel aspect related to the world of HA, histaminergic receptors, their activation and intracellular pathways, and their interactions with other mediators. This knowledge represents a step forward in the study and characterization of new histaminergic molecules for the treatment of human diseases.
